# Quantification of Visual Fixation Behavior and Spatial Orientation Memory in *Drosophila melanogaster*

**DOI:** 10.3389/fnbeh.2019.00215

**Published:** 2019-09-13

**Authors:** Hung-Hsiu Yen, Rui Han, Chung-Chuan Lo

**Affiliations:** ^1^Institute of Systems Neuroscience, National Tsing Hua University, Hsinchu, Taiwan; ^2^Institute of Bioinformatics and Structural Biology, National Tsing Hua University, Hsinchu, Taiwan; ^3^Brain Research Center, National Tsing Hua University, Hsinchu, Taiwan

**Keywords:** Buridan's paradigm, fruit fly, landmark orientation, orientation memory, working memory, visual fixation

## Abstract

*Drosophila Melanogaster* has been shown to exhibit short-term orientation memory by fixating on orientations toward previously displayed visual landmarks. However, the fixation behavior varies and is often mixed with other types of movement. Therefore, carefully designed statistical measures are required in order to properly describe the characteristics of the fixation behavior and to quantify the orientation memory exhibited by the fruit flies. To this end, we propose a set of analytical methods. First, we defined the deviation angle which is used to quantify the deviation of the fruit fly's heading from the landmark positions. The deviation angle is defined based on the fruit fly's perspective and is able to reveal more task-relevant movement patterns than the commonly used definition which is based on the “observer's perspective.” We further introduce a temporal deviation angle plot which visually presents the complex movement pattern as a function of time. Next, we define the fixation index which tolerates fluctuation in the movement and performs better in quantifying the level of fixation behavior, or the orientation memory, than the conventional method.

## Introduction

Visual pattern fixation is an innate behavior of many insects and is characterized by persistent movement toward a visually salient landmark in the environment. In *Drosophila melanogaster*, such behavior has been well-demonstrated by Buridan's paradigm. Due to the innateness and robustness of visual pattern fixation, it has been used to test various cognitive functions such as visual and special working memory, and locomotive control (Powell and Dobzhansky, 1976; Götz, 1980; Goetz, 1989; Menzel et al., 1996; Strauss, 2002; Pan et al., 2009; Lin et al., 2013; Paulk et al., 2013).

In the traditional Buridan's Paradigm, a drum like arena is surrounded by fluorescent light tubes that illuminate the wall of the arena. Two vertical black strips, serving as the visual landmarks, are attached on the inner side of the wall and are separated by 180°. A circular stage surrounded by a water moat is located at the center of the arena and a wing-clipped fly is allowed to freely move on the stage. Without any training, a naïve fly tends to move back and forth between the landmarks and exhibits a persistence fixation. During the experiment, the fly's position is recorded and converted into deviation angles toward each of two landmarks (Strauss and Pichler, 1998; Colomb et al., 2012).

In order to further study *Drosophila*'s memory about the landmarks, scientists have to control the appearance and the position of landmarks. Early experiments use cylinder mechanics to move a landmark, but as the technology developed, LEDs replaced the analog ones because of LEDs fast response time and high refresh rate. With this powerful LED display technology, neurobiologists are able to design more intricate behavioral experiments (Strauss et al., 1997; Maimon et al., 2008; Reiser and Dickinson, 2008; Ofstad et al., 2011; Maisak et al., 2013).

To quantify the behavior of fruit flies in these types of tasks, several indices have been used to measure the level of memory retained after the landmarks disappeared. For example, walking distance toward disappeared landmarks or location distribution of fruit flies in the circular plate before and after the landmark offset. However, we discovered that these indices were not efficient in quantifying the difference between flies that retained memory and flies that did not due to the highly diverse and variable nature of the movement of fruit flies. Besides, most studies focused on analyzing *Drosophila*'s “group” behavior and less were on the “individual” behavior (Strauss and Pichler, 1998; Neuser et al., 2008; Xiong et al., 2010).

In the present study, we improved the previous methods by first changing the way how angular deviation is calculated from observer's perspective to fruit fly's perspective. Compared to the traditional method, our method demonstrated an advantage on recognizing fly's clockwise or counterclockwise movement pattern. Next, we introduced a new type of plot to visualize the movement of single fruit fly and a fixation index that can better quantify the quality of the fixation behavior. We applied this method in a 3-stage spatial orientation memory task based on the Buridan's paradigm and demonstrated that our method could successfully quantify the fixation behavior and fruit fly's short-term memory about the landmark orientation.

## Materials and Methods

### Animal Preparations

Two to three days old female fruit flies (*Drosophila melanogaster*) (Colomb et al., 2012) (Canton-S) were used in the present research. The flies were incubated in an environment with a temperature between 24°C and 25°C and a humidity between 60 and 70%. To keep the flies from flying away during the behavioral task, the wings of the flies were clipped under cold anesthesia 3–5 days prior to the task. The wing-clipped flies were put separately in the test tubes that were loaded with food prepared based on Nutri-Fly™ Bloomington Formulation 66–112. To maximize the behavioral performance, the flies were kept food-deprived prior to the experiments by moving them to the tubes loaded only with agar 24 h before the experiment.

### The Behavior Arena

**Figure 1:**
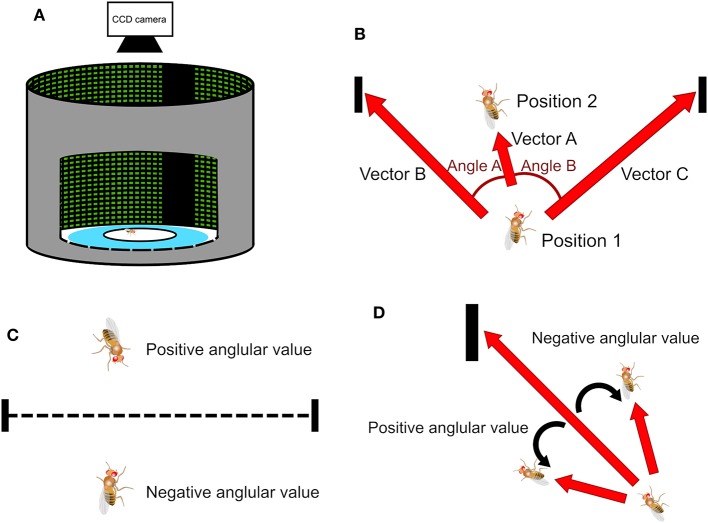
The experimental setup of the spatial orientation memory task and methods of deviation angle calculation. **(A)** The task is designed based on the Buridan's paradigm. A wing-clipped fruit fly is placed on a circular platform in an arena surrounded by an LED display. During the stimulus stage two vertical black strips, separated by 180° are displayed on the screen serving as the landmarks. **(B)** When a fly moves from Position 1 to Position 2, it creates a vector A. Position 1 is associated with two vectors, B and C, and each vector points to one of the two landmarks. We can obtain two angles, one between vectors A and B and the other between vectors A and C. **(C)** In a traditional method, called the observer's perspective in this paper, the sign of the angles is determined by whether the fly is located above or below the midpoint line, which is the line connecting the center points of the two strips. **(D)** In the present study, the sign of the angles is determined by the sign of the cross product of two associated vectors. We call this method the fruit fly's perspective.

To perform the spatial orientation memory task, we constructed a circular arena. The arena has a diameter of 26 cm with an elevated platform of 8.5 cm in diameter located at the center of the arena (Figure 1A). The fruit flies were only allowed to stay on the platform and the space between the platform and the edge of the arena was filled with water. The arena is surrounded by a 360° LED display which is controlled by a computer through an Arduino board.

### Spatial Orientation Memory Task

We demonstrated our methods in a fruit fly behavioral task modified from the classic Buridan's paradigm (Götz, 1980; Strauss and Pichler, 1998). The task was designed to test whether a fruit fly was able to maintain short-term memory about the locations of visual landmarks after they disappeared. In the task, two dark stripes, one at 0° and the other at 180°, were presented on the LED display and they served as the visual landmarks (Figure 1A). The landmarks induced an innate visual fixation behavior in which a fruit fly walked back and forth between the two landmarks repetitively. We performed two versions of the task. In the first version, a fruit fly was placed on the platform with the presence of the visual landmarks and it took an average of 60–80 seconds for a fly to establish a stable fixation behavior. After several rounds of persistent movement between the landmarks were observed, we switched off the landmarks at the moment when the fly crossed the midline and then we started to record the fly's response. The midline was an imaginary line (not displayed on the platform) that lay between 90° and 270°, and separated the platform into two equal parts.

The second version of the task composed of three 90s-stages and a fly was allowed to freely move on the platform in each stage. In the stage 1 (the pre-stimulus stage), all LEDs were turned on with no visual landmark presented on the display. In the stage 2 (the stimulus stage), two landmarks were displayed as described above. In stage 3 (the post-stimulus stage), the landmarks were removed so that the stimulus setup is identical to that of the pre-stimulus stage. The behavior in the post-stimulus stage was compared with that in the pre-stimulus stage. Any behavioral difference characterizes a trace of memory about the visual landmarks which were only presented in the stimulus stage.

### Recording, Sampling, and the Generation of Walking Trajectories

Movement tracking was performed by a Python script using OpenCV. A fly appeared as a dark spot on the white platform in the images taken by the CCD camera (Guppy Pro from Allied Vision Technology). The images were recorded with a sampling rate of 20 Hz, and converted into grayscale. The images were then inverted, so that the fly was represented by a white spot in the images. In order to enhance the trackability of the white spot, we applied the Gaussian Blur to enhance the appearance of white spot. The exact location of the fly was represented by the x-y coordinates of the pixel with the largest intensive in the white spot. The location of the fly in each image were recorded during the experiment and the movement trajectories were generated from the recording after each trial.

### Simulated Walking Trajectories

To precisely understand how the movement pattern affects the outcome of different measurements of movement, we generated simulated walking trajectories with specific patterns. The trajectories were generated based on the polar coordinates with the radius and angle as the variables. We created five movement patterns: (1) a straight walk between two landmarks on the midpoint line, (2) a straight walk between two landmarks, but 2 cm off the midpoint line, (3) a walk along the edge in the clockwise direction, (4) a walk along the edge in the counterclockwise direction, and (5) a random walk. The random walk was composed of pattern (3) and pattern (4) randomly with varying radius. Before the pattern was determined, the number of points, or the duration, for each pattern was generated randomly based on multiples of 10 between 10 and 100. We then decided the variation of radius randomly from three scenarios: increasing by 0.04 cm gradually, decreasing by 0.04 cm gradually, and remaining unchanged. To mimic the stochastic nature of the real trajectories of fly walking, we added noise to the radius variable of the trajectories with a standard deviation (σ), which was set to 0.4 cm, 0.4 cm and 0.08 cm for the patterns (1), (2), and (5), respectively. No noise was added in the patterns (3) and (4), because when real flies walked along the edge, the fluctuation of trajectories were so small that they could be treated as a perfect circle. To be consistent with the sampling rate of the camera used to record the real trajectories of fruit flies, we generated the simulated trajectories with a frequency of 20 Hz.

## Results

### Angular Deviation

In a Buridan-type of task, a central question is whether the fruit fly moves toward a visual landmark, or a specific orientation. This is done by calculating the deviation angle between the moving direction of the fly and the direction of the landmarks (Figure 1B). Previous studies (Phillips et al., 2005; Triphan et al., 2010; Riemensperger et al., 2011; Webb and Wystrach, 2016) proposed that one can first calculate the inner products between the unit vector representing the fly moving direction (Vector A in Figure 1B) and the unit vectors that represents the directions of the landmarks with respect to the fly (Vectors B and C in Figure 1B). Two deviation angles (Angles A and B) can be quickly obtained by applying an inverse trigonometric function (arccosine) to the inner products. Then, one only uses the smaller of the two angles because it represents the deviation angle from the landmark which the fly is approaching. The value of the angle calculated this way is always positive. The sign of the angle is determined by the fly's position relatives to the midpoint line, a line that connects the midpoints of both landmarks (dashed line in Figure 1C). The angle is set to be positive if a fly is above the line, while negative if below (Figure 1C). We call such definition “observer's perspective” because the dividing line is defined by the observer and walking on either side of the line does not make any difference to the fly, considering that the platform is symmetric along the line. We propose a different method to calculate the deviation angle. First, we calculate the cross product of the unit vector of the fly movement direction and the unit vectors that represent the directions of the two visual landmarks (Figure 1D) and an inverse trigonometric function is applied to compute the angle between the two vectors. The cross product is done for both visual landmarks and one that yields the smallest absolute value is chosen because it indicates the landmarks which the fly is approaching. The sign of the angle is naturally determined by the cross product. Positive angle represents that the fly is moving toward the left side of the landmarks while negative angle indicates the right side. We call such definition fruit fly's perspective (Strauss and Pichler, 1998) because the sign of the deviation angle is defined by how the fly approaches the landmarks. As we shall see below, this definition is particularly useful for indicating a common type of movement of flies in the arena.

### Histogram of Deviation Angle

**Figure 2:**
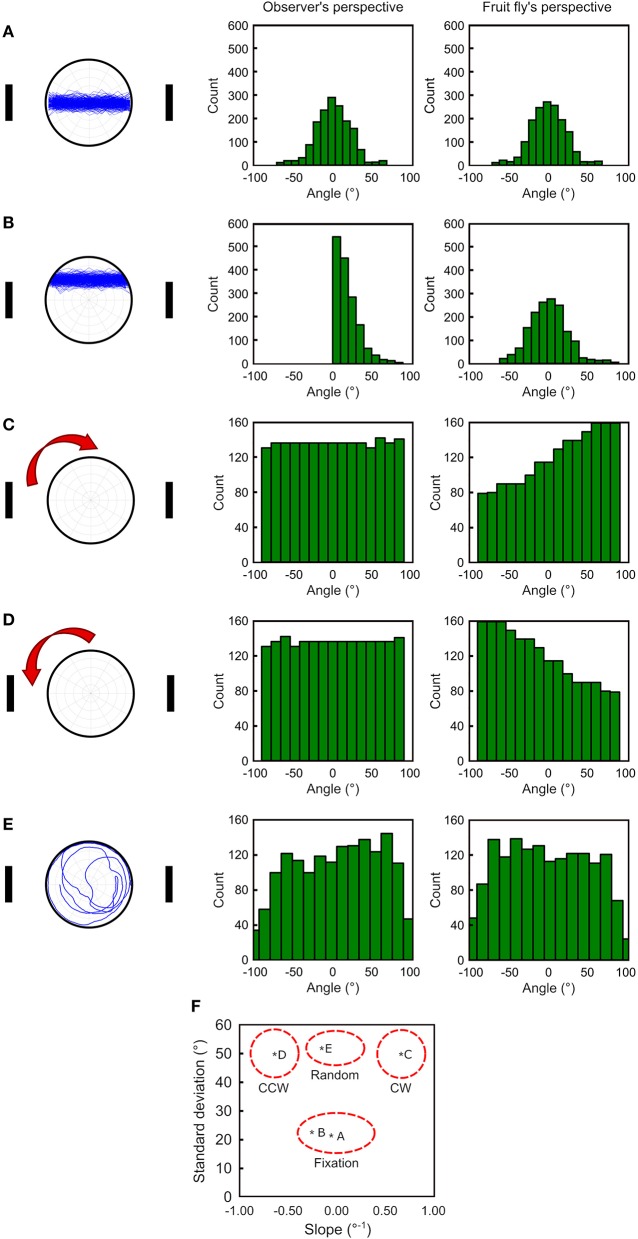
To compare the effectiveness in quantifying the movement patterns between the observer's perspective and the fruit fly's perspective. We generate the simulated walking trajectories in five different patterns (left column) and plot the deviation angle histograms for the observer's perspective (middle column) and the proposed fruit fly's perspective (right column) as a way to quantify the movement behavior. **(A)** For movement between the two landmarks, the two methods produce similar histograms. **(B)** For movement between the two landmarks but with a slight shift from the midpoint line, the observer's perspective produces an asymmetric histogram peaking at 0, while fruit fly's perspective remains the same as in **(A)** (right). **(C,D)** For clockwise and counterclockwise movement along the edge, the observer's perspective produces similar histograms with a flat distribution, while the fruit fly's perspective produces distinct distributions between the two movement directions. **(E)** For random walks, both methods produce histograms with a similar distribution. **(F)** By calculating the standard deviation and the slope between ±50° for each histogram, one can classify the movement patterns using a 2-D plot with the two measures on each axis. Movements with strong fixation are represented by the lower middle part of the plot and random movements are in the upper middle part of the plot. Moving along the edge with the clockwise (CW) or counterclockwise (CCW) directions takes the upper right or upper left part of the plot, respectively.

Deviation angle histograms are often used for analyzing the fixation behavior of fruit flies. To test whether our fruit fly's perspective has any advantages in analyzing the walking behavior, we generate four simulated movement patterns (see Methods): (1) between two landmarks on the midpoint line (Figure 2A left), (2) between two landmarks above the midpoint line (Figure 2B left), (3) clockwise along the edge (Figure 2C left), (4) counterclockwise along the edge (Figure 2D left), and (5) random walk (Figure 2E left). We compare the two methods based on observer's and fruit fly's perspective using the deviation angle histograms. For the pattern 1, both methods produced nearly identical histograms (Figure 2A middle and right). For the pattern 2, the observer's perspective produces an asymmetric histogram while the fruit fly's perspective produces a histogram that is similar to that in the pattern 1 (Figure 2B middle and right). Therefore, as long as a fly walks back and forth between the visual landmarks, no matter whether it walks perfectly along the midpoint line or on the side of the line, our method gives rise to a deviation angle histogram with a roughly symmetric shape and with a peak at 0°. In contrast, the traditional method is very sensitive to whether the fly walks right on the midpoint line and produces a distinct histogram if it is not.

Next, we investigate how the two methods perform when a fly walks along the edge, a pattern commonly observed in fruit flies. We discover that the traditional method cannot detect whether the fly walked clockwise or counterclockwise. Moreover, the histograms are flat, which is indistinguishable from a random walk (Figures 2C–E middle). In contrast, our method produces a monotonically increased or decreased histogram for the clockwise or counterclockwise movement patterns, respectively, and both histograms are distinct from the random walk, which gives rise to a flat histogram (Figures 2C–E right).

We further quantify the shape of the histograms and propose indices that can be used to classify the movement patterns of fruit flies. We first calculate the standard deviation σ of the histogram and find that if a fly performs fixation behavior (Figures 2A,B right), the standard deviation is approximately half of that for the movement along the edge (Figures 2C,E right). Next, we calculate the slope of the histogram between the range from −50° to +50°. Because the clockwise or counterclockwise movement patterns produce a monotonically increasing or decreasing histogram, the slope is a large positive or negative value, respectively. In contrast, the histograms from random walks or from fixated movement have relatively small slopes. By plotting the two indices (standard deviation and slope) on a two-dimensional plot, we can clearly distinguish the movement patterns of the fruit flies (Figure 2F).

### Temporal Deviation Angle Plot

**Figure 3:**
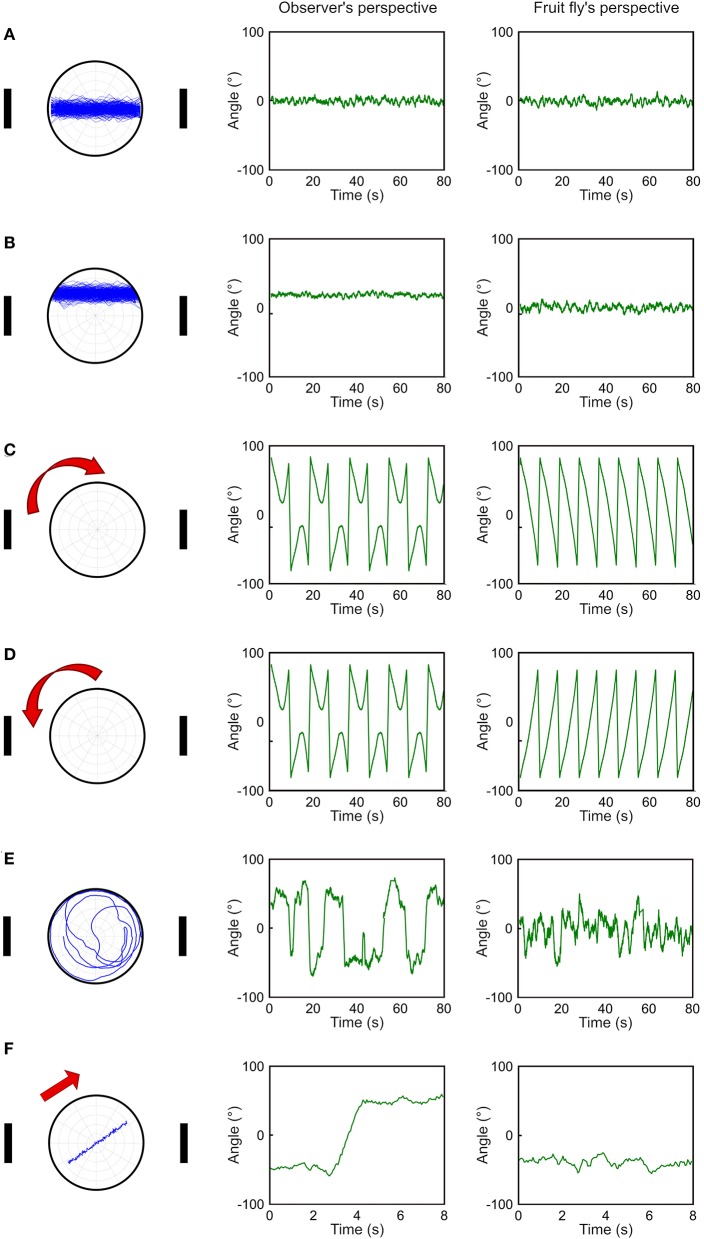
Comparison between observer's perspective and fruit fly's perspective in temporal deviation angle plots. **(A–E)** We compare five different movement patterns (left column) as in this figure for the observer's perspective (middle column) and the fruit fly's perspective (right column) by plotting the deviation angle as a function of time. The fruit fly's perspective can clearly distinguish the clockwise movement from the counterclockwise movement **(C,D)**. **(F)** The two perspectives give rise to the most distinct curves when the fly moves across the midpoint line. This movement produces a jump in the curve based on the observer's perspective and the jump could lead to a misperception that the fly made a drastic movement change.

Although the deviation angle histogram is useful, it only provides information about the overall movement pattern of a fly over a large period of time and does not reveal momentary movement types. To address this issue, we propose a temporal deviation angle plot which shows the deviation angle as a function of time. We first demonstrate this plot using the same simulated walks presented in Figure 2. Due to fluctuations in trajectories, we smooth the trajectories by adopting moving average with a time window of 20 data points (equivalent to 1 s). When a fly walks between two landmarks along the midpoint line (Figure 3A), or in the upper part of the plate (Figure 3B), both methods produce a similar pattern with overall constant deviation angles. The major difference is that in the case of walking in the upper part of the plate, the method based on observer's perspective gives rise to positive values instead of values around zero as produced by our method (Figure 3B). When the simulated fly walks along the edge in the clockwise or counterclockwise direction (Figures 3C,D), we can observe oscillatory patterns of the deviation angles with both methods. However, the method based on the observer's perspective does not distinguish between the two opposite movement patterns. In contrast, our method gives rise to the angular deviation that clearly indicates the different moving direction. For random walk, both methods produce plots that are distinct from all other movement patterns (Figure 3E). However, the observer's perspective led to a curve which fluctuates in a wider range than the fruit fly's perspective does. This difference is mainly due to the way how the sign of the deviation angle is defined in the two methods. When a fly moves across the midpoint line, the sign changes in observer's perspective, leading to a jump in the curve, while the fruit fly's perspective gives rise to a much smoother curve (Figure 3F). The plot based on the observer's perspective can lead to a misperception that the fly made a drastic change in its movement pattern. However, this is not the case and the actual movement trajectory is pretty smooth. In the fruit fly's perspective, a jump in the curve is always associated with a rapid turn that changes the approached landmark from one to the other, or that shifts the approached landmark from one side of the visual field to the other side. Both are important indicators for the behavioral change of fruit flies. However, in the observer's perspective, such a jump in the curve may be produced by a small step that crosses the midpoint line and may not indicate any significant behavioral change.

### Analyzing the Deviation Angle Before and After the Landmarks Offset

**Figure 4:**
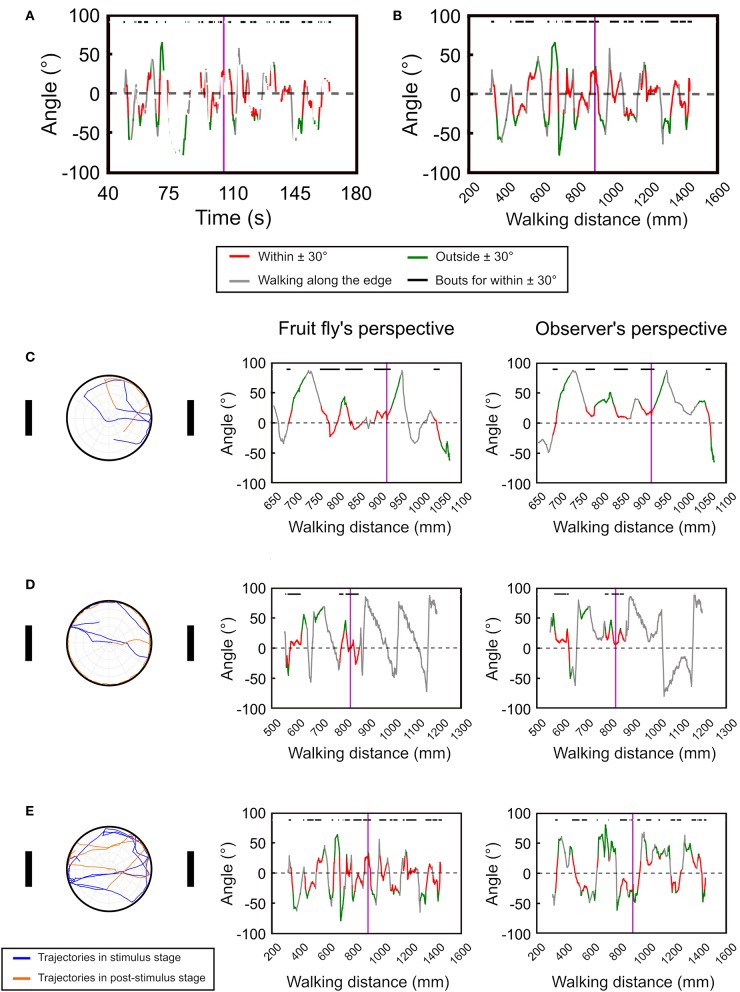
The temporal deviation angle plot can be improved by plotting the angle as a function of walking distance instead of time. **(A)** For movement of real flies, plotting the deviation angle as a function of time produces a disconnected curve due to the frequent pauses of the flies. **(B)** The issue can be resolved by plotting the deviation angle as a function of moving distance. The vertical purple line indicates the offset of the landmarks. The red segments represent the movement bouts that have the deviation angles ≤ ±30° while the green segments are for ≥ ±30°. The gray segments indicate bouts of movement that is along the edge of the platform. The black bars on top of the plots denote the bouts corresponding to the red segment. Using the temporal deviation angle plot, we identify three major types of movement patterns after the landmarks offset. Here we show one example for each pattern. **(C)** The fly immediately disengages from the fixation behavior after the landmark offset. **(D)** The fly continues the fixation behavior after the landmark offset, but disengages from the fixation behavior once it reaches the edge of the platform. **(E)** The fly continues the fixation behavior for a long period of time. The left column shows the moving trajectories while the middle and right column displays the temporal deviation angle plot in the fruit fly's and the observer's perspectives, respectively. In the trajectory plots (left column) the trajectories before and after the landmark offset are represented by the blue and orange curves, respectively.

Next, we test the temporal deviation angle plot with real data from the version 1 of the spatial orientation memory task (Figure 4, see Materials and Methods). In order to smooth the movement trajectories, we plot the moving average of the deviation angle with a time window of 20 data points, or 1 s. Compared to the simulated data as shown in Figure 3, the fruit flies exhibited intermittent rest bouts during the experiment, which yields discontinued lines in the plot (Figure 4A). The discontinuity makes the analysis difficult. To address this issue, we changed the x-axis from time to walk distance (Figure 4B). To convey more information in the plots, we use red line to represent the deviation angles with absolute values that are smaller than 30°, and green line for the values that are larger than 30°. We also use gray to indicate the periods when the fly moved along the edge of the platform. To further visualize the periods in which a fly exhibit landmark approaching behavior, or the red bouts, they are indicated by black horizontal bars at the top of the plots.

Using the temporal deviation angle plots, we discover that fruit flies behavior immediately following landmark offset can be categorized into three major patterns. First, the flies quickly disengage from the fixation behavior after the landmark offset (Figure 4C left and middle). Second, the flies continue their fixation behavior by walking toward a landmark until they reach the edge, and start to walk along the edge afterwards (Figure 4D left and middle). Third, the flies maintain the fixation behavior for a long period of time by walking between the two landmarks (Figure 4E left and middle). These different movement patterns are clearly visible on the angle-time plots. For comparison, we also display the deviation angle plots based on the observer's perspective (right column in Figure 4). For the fly that moves along the edge as in Figure 4D, one cannot tell whether the fly moves in the clockwise or counterclockwise direction from the plot that is based on the observer's perspective (Figure 4D right). But the plot based on the fruit fly's perspective clearly indicates a clockwise movement (Figure 4D left).

### The Fixation Index (FI)

**Figure 5:**
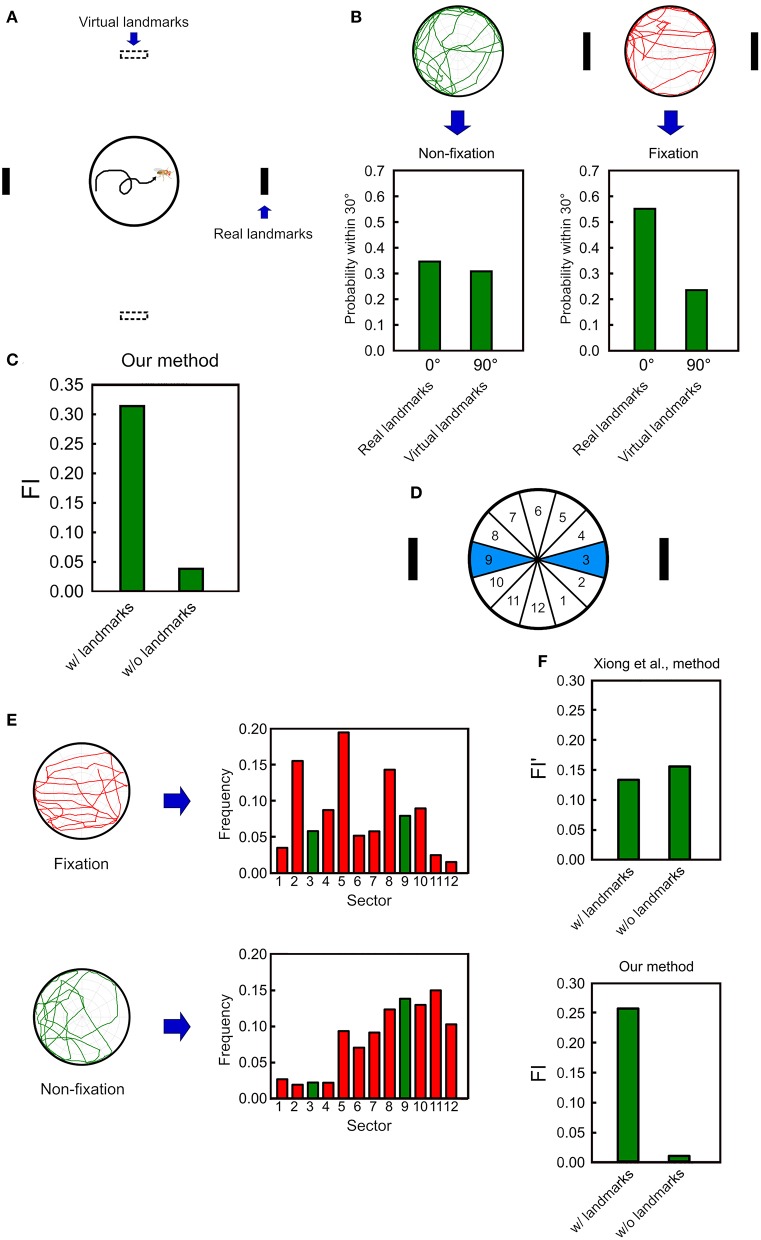
The fixation index (FI) as an effective indicator of the fixation behavior. **(A)** We first calculate the probabilities of the deviation angle falls within 30° from the center of the real landmarks (solid black strips) and from the center of the virtual landmarks (hollow black stripes), which are 90 degrees from the real landmarks. (B) For the non-fixation movement pattern (before landmark onset), the two frequencies are nearly the same, while for the fixation movement pattern (after landmarks onset), the two frequencies are significantly different. **(C)** The FI is calculated by subtracting the frequency for the virtual landmarks from that for the real landmarks. The FI is significantly higher when the landmarks are presented than when they are not. **(D)** In Xiong et al. (2010), the fixation behavior is quantified by calculating the frequencies that a fruit fly is located in between the two wedges which correspond to the direction of the landmarks. **(E)** The frequency histograms for the 12 wedges defined in Xiong et al. (2010) for fixation (top) and non-fixation (bottom) movement patterns. **(F)** The FI calculated from Xiong et al. (2010) does not exhibit a significantly higher value for the fixation than for the non-fixation behavior, while our method does.

To further quantify the overall performance of a fly in the spatial orientation task, we need a measure to describe the relative frequency of the fly's walk between the two landmarks with respect to a reference direction. To this end, we create a pair of virtual landmarks that are 90 degrees from the real landmarks (Figure 5A). Next, we calculate the probabilities that the deviation angle falls within 30 from the center of the real landmarks or from the center of the virtual landmarks. The difference between these two probabilities serve as a measure for the fixation behavior of the fruit flies. Without any landmark, a fly does not fixate on any direction and the two probabilities are expected to be close to each other (Figure 5B left). In contrast, when the landmarks are displayed, a fly exhibits the fixation behavior on the landmarks, yielding a much higher probability for the real than for the virtual landmarks (Figure 5B right). Based on the observation, we define a fixation index (FI) which is the difference between the two probabilities. A positive value which is considerably higher than zero indicates the fixation behavior while a non-fixation behavior yields a value which is close to zero (Figure 5C). Note that whether a positive and non-zero FI is statistically significant should be tested at the group level.

We compare our FI with a similar measure previously used in Xiong et al. (2010), in which the platform was divided into 12 wedges. Based on this study, the fixation index (FI′) can be defined as the sum of the frequencies of a fruit fly located in the two (3 and 9) wedges that match the directions of the landmarks (Figure 5D). It is intuitive to expect that the fixation index is larger when the fly fixates than when the fly does not fixate on the landmarks. However, a fruit fly rarely moves in a perfect straight line through the center of the platform. Therefore, even a significant fixation behavior still yields large non-zero values of the frequencies for all wedges. It turns out that when the fixation behavior is not perfect, such a measure is not effective in distinguishing between the fixation and the non-fixation behavior, while our FI shows a significant difference between the two behavioral patterns (Figures 5E,F).

### FI in a Spatial Orientation Working Memory Task

In addition to calculating FI for each stage of the task, we suggest that one should also calculate the momentary FI and observe how the fixation behavior changes in time. To this end, we design a three-stage task of spatial orientation memory (see Materials and Methods) and plot a temporal FI plot. Because the stimulus setup (no landmark) in the pre-stimulus stage is identical to the post-stimulus stage, the observed behavior differences between the two stages can be contributed to the influence from the landmarks presented in the stimulus stage. In other words, the memory trace can be identified by comparing the FIs of the pre-stimulus stage and stimulus stage.

**Figure 6:**
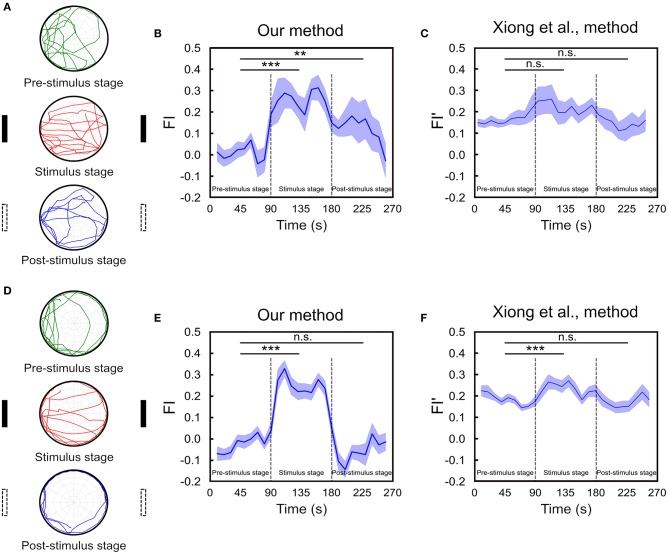
The spatial orientation memory can be identified using FI based on our method. **(A)** The experiment consists of three 90s stages and a fly is allowed to freely move on the platform. Two landmarks are displayed during the stimulus (second) stage while not in the pre- and post-stimulus stages. Movement trajectories in the post-stimulus stage are compared to those in the pre-stimulus stage for the trace of spatial orientation memory. The trajectories of one representative trial of a fly that exhibits strong fixation behavior are displayed here. **(B)** The FI is calculated based on our method for each of the 10 s time window through the entire trial for the group of flies that exhibit strong fixation behavior in the post-stimulus stage. The FI in the post-stimulus stage is significantly higher than that in the pre-stimulus stage (*n* = 18, **p < 0.01, ***p < 0.001), indicating the presence of spatial orientation working memory. **(C)** The FI is calculated based on the Xiong et al. (2010) method (*n* = 18, n.s. means no significant) for the same group of flies shown in **(B)**. **(D)** Same as in **A** but for a fly that did not exhibit clear fixation behavior during the post-stimulus stage. **(E)** Same as in B but for the group of flies with no fixation behavior during the post-stimulus stage. Student *T*-test shows the difference between pre- and post-stimulus stage is non-significant (*n* = 47, ***p < 0.001). **(F)** Same as in E but the FI is calculated based on the Xiong et al. (2010) method (*n* = 47, ***p < 0.001).

We perform the task for 65 fruit flies. In order to compare the temporal FI plots between flies that maintain working memory of the landmark direction and flies that do not, we classify the flies into two groups. This is done by calculating the difference between the probabilities that an individual fly move toward the vicinal regions (±52.5°) around the landmark positions (0° and 180°) and that the fly move toward the perpendicular directions (±30° around 90° and 270°). We calculate the difference for each fly and choose those (*n* = 18) that are one standard deviation above the group mean for the first group and leave the rest (*n* = 47) in the second group. We plot the index as a function of time for both groups of fruit flies (Figure 6). For the first group, we observe large FIs during the stimulus stage and the FI drops during the post-stimulus stage but is still considerably higher than those in the pre-stimulus stage. Interestingly, the FI gradually decays until it reaches zero during the post-stimulus stage (Figures 6A,B). To check the significance of the differences in FIs between stages, we calculate the mean FI of each fly in each stage and apply the multi-factor within group ANOVA test to examine their difference. The result indicates that although the FI gradually drops in the post-stimulus stage, the difference between post- and pre-stimulus stages is significant (*p* < 0.01). We compare our FI with that (FI′) used in Xiong et al. (2010) (Figure 6C). We do not observe any significant difference in FI′ between the three stages (*p* = 0.10 between pre-stimulus stage and stimulus stage. *p* = 0.86 between pre- and post-stimulus stage), although the fixation behavior can be identified visually.

On the other hand, for the group of flies that does not exhibit clear fixation behavior during the post-stimulus stage, the FI of this stage is not significantly different from that of the pre-stimulus stage (*p* = 0.23) (Figures 6D,E). The same result is obtained from FI′ based the method in Xiong et al. (2010) (*p* = 0.73) (Figure 6F). Note that although in both methods the fixation index in the stimulus stage is significantly higher than that in the pre-stimulus stage (*p* < 0.001) as expected, the difference between the two stages is much smaller in the Xiong et al. (2010) method than in ours, suggesting that the former is less sensitive to the actual fixation behavior.

## Discussion

In the present study, we propose three analytical tools for fruit fly's fixation behavior and short-term memory of spatial orientation. We first define the deviation angle based on cross product of the fly's heading direction and the landmark direction. The sign of the angle is determined by the position of the visual landmark with respective to the heading direction of the fly, and hence carries the information of the location of the visual landmark in the fruit fly's perspective, which is important for the interpretation of the fly behavior. Another advantage is that the deviation angle is sensitive to the direction of circling movement. Circling along the edge of the platform is a prominent behavior exhibited by the flies and characterizing the movement direction help with understanding their preferences.

We next introduce the temporal deviation angle plot which reveals the temporal information of the deviation angle. This helps us with inspecting the temporal evolution of the deviation angle throughout a trial, and most importantly, how flies change their movement patterns in response to changes in the stimuli. The plot is much more efficient than the typical deviation angle histograms used in many earlier studies.

Finally, we proposed the fixation index (FI) for quantifying fixation behavior of the fruit flies. Using the deviation angle instead of the wedged zones as in the previous studies, the FI tolerates the fluctuation and randomness in the real movement of flies and can still captures the tendency of fixation behavior even when it is not perfect.

In the present study we only consider the moving direction of the flies when calculating FI. The orientation of the flies during rest is not detected due to the insufficient resolution of the camera. Since a large number of rest bouts are usually observed in a trial, it is interesting to analyze the orientation during rest and potentially consider it as a part of the fixation behavior. We plan to implement high resolution cameras and monitor the resting behavior in future studies.

In an earlier study, Strauss and Pichler (1998) determined the degree of fixation after landmark offset by measuring how long a fruit fly continued its fixation behavior before breaking away. However, we found that even after the fruit fly broke away from the fixation behavior, it could be resumed later. Such return of fixation has large inter-individual variability but can be identified by our methods. Specifically, the presence of orientation memory during the post-stimulus stage can be indicated by the periods in which the FI is significantly larger than that in the pre-stimulus stage. For the group of flies which exhibit strong fixation behavior during the post-stimulus stage, the FI remains high for the first ~50s, but gradually decays afterwards (Figure 6B). This is consistent with the short time-span and temporal decay natures of the working memory. Therefore, we suggest that FI provides a good indicator for spatial orientation working memory of landmark orientation in the present task.

In conclusion, our methods provide accurate and versatile measures for quantifying the fixation behavior of insects, and lead to the identification of fixation activity which persists for much more than a few seconds after landmark offset as previously reported. Therefore, our methods create the possibility of studying orientation memory in a much longer time frame using Buridan's or other more complex spatial orientation tasks.

## Data Availability

The data and code used in this study are available upon request to the corresponding author.

## Author Contributions

H-HY constructed the behavior arena, carried out the experiment, analyzed the data, and wrote the manuscript. RH wrote the manuscript and made the figures. C-CL designed the study and wrote the manuscript.

### Conflict of Interest Statement

The authors declare that the research was conducted in the absence of any commercial or financial relationships that could be construed as a potential conflict of interest.
